# Effects of dance therapy on cognitive and mental health in adults aged 55 years and older with mild cognitive impairment: a systematic review and meta-analysis

**DOI:** 10.1186/s12877-023-04406-y

**Published:** 2023-10-26

**Authors:** Chen-shan Huang, Yuan-jiao Yan, Yu-ting Luo, Rong Lin, Hong Li

**Affiliations:** 1 School of Nursing, Fujian Medical University, No. 1 Xueyuan Road, Shangjie Town, Fuzhou City, 350122 Fujian Province China; 2 Department of Nursing, Fujian Provincial Hospital & Shengli Clinical Medical College, No. 134 Dongjie Street, Gulou District, Fuzhou City, 350001 Fujian Province China

**Keywords:** Dance therapy, Mild cognitive impairment, Older adults, Cognitive, Mental health, Meta-analysis

## Abstract

**Background:**

Individuals with mild cognitive impairment are at high risk of developing dementia. Dance therapy has promising applications in delaying cognitive decline. However, the effectiveness of dance therapy for older adults with mild cognitive impairment is unclear. The objective of this review was to evaluate the effectiveness of dance therapy on global cognitive function, specific cognitive subdomains, quality of life, and mental health in older adults with mild cognitive impairment to enrich health management strategies for dementia.

**Methods:**

Electronic databases and grey literature were searched from inception up to September 23, 2023. The language was limited to English and Chinese. Relevant studies were screened and assessed for risk of bias. A meta-analysis and subgroup analyses stratified by measurement instrument, dance type, intervention duration, and frequency were conducted using the STATA 16.0 software. This review was conducted in accordance with the PRISMA guidelines.

**Results:**

Ten studies involving 984 participants aged 55 years and over who met the eligibility criteria were included. Dance therapy significantly improved global cognitive function, memory, executive function, attention, language, and mental health (i.e., depression and neuropsychiatric symptoms). However, the effects of dance therapy on processing speed, visuospatial ability, and quality of life in older adults with mild cognitive impairment remain inconclusive. Moreover, dance interventions of longer duration (> 3 months) improved global cognition more than shorter interventions.

**Conclusion:**

This review reported that dance therapy was effective in improving global cognitive function, memory, executive function, attention, language, and mental health (i.e., depression and neuropsychiatric symptoms). Hence, it may be an effective non-pharmacological complementary treatment for older adults with mild cognitive impairment.

**Supplementary Information:**

The online version contains supplementary material available at 10.1186/s12877-023-04406-y.

## Background

Rapid global aging leads to a dramatic increase in the prevalence of age-related cognitive impairment. Research shows that the number of older individuals with dementia is increasing globally and is expected to rise to 152 million by 2050 [1]. China has the largest number of individuals with dementia, accounting for approximately 25% of the total global dementia population; this places a heavy economic and social burden on the public health system [2].

Given the irreversible course of dementia and the limitations of treatment, there is a growing interest in early preventive interventions. Mild cognitive impairment (MCI) is a transitional condition between normal aging-related cognitive decline and dementia, with a high probability for regression to dementia [3]. According to data, the incidence of MCI among adults aged ≥ 60 years in China is 15.54%, and the number of patients is 38.77 million [4]. Considering that MCI puts individuals at high risk for developing dementia, early intervention at this stage may be effective in preventing or slowing the progression to dementia. Due to the limited efficacy of the currently available pharmacological treatments for MCI and the presence of certain drug side effects [5], non-pharmacological treatments aimed at delaying disease progression and improving quality of life have become a hot topic of research.

A series of studies have reported the potential benefits of non-pharmacological cognitive interventions in improving cognitive function and promoting mental health [6, 7]. The provision of non-pharmacological care to individuals with MCI may be a good approach. Despite the rapid increase in various types of non-pharmacological interventions over the past few years, adherence to interventions among older adults with MCI appears to be poor and there is significant variability between individuals [8, 9]. Possible reasons affecting the adherence include lack of motivation to participate, low interest, single form of intervention, and repetition [8]. Therefore, non-pharmacological interventions that are enjoyable and fun are more likely to improve adherence among individuals [10]. A growing body of evidence suggests that dance therapy (DT) appears to be a better intervention and has shown significant benefits in terms of improving cognitive function and other health-related outcomes [7, 11, 12]. DT is a physical and mental activity in which the body moves purposefully and rhythmically to music and is often considered an enjoyable form of multimodal activity [13]. This physical and cognitive practice associated with music can motivate individuals, channel emotions, trigger memories, and avoid boredom, thus creating an interest in continuing the practice [14]. The pleasure and enjoyment experienced by individuals through dance increases the likelihood of regular participation and adherence, which may be essential in achieving long-term health benefits [15].

DT induces and has long-term positive effects on neuroplasticity, such as learning and remembering complex motor movements, focusing attention on following instructions, executing complex movement patterns, integrating visual and rhythmic movements, and social cognition that links emotional expression between individuals in social interactions [16]. DT is also fundamentally a social activity with proven benefits in promoting social participation in older adults with MCI, which may also be associated with better cognitive outcomes [16]. Emerging evidence suggests that dance-based interventions are less expensive to implement, easy to apply in different settings (e.g., clinics, community centers, and nursing homes), and promote physical and mental health in older individuals in a variety of settings [17]. Hence, DT may be a powerful intervention that can be easily promoted to older individuals with MCI as part of a healthy lifestyle.

DT may help maintain or improve cognitive function and reduce the risk of developing dementia over time. However, existing systematic reviews of DT have reported inconsistent results regarding improvements in cognitive function [7, 11, 12]. Furthermore, there is limited examination of key issues, such as mental health, which have been strongly associated with quality of life in individuals with MCI [18]. Therefore, we conducted a systematic review and meta-analysis of all available relevant evidence (including published and grey literature) to examine the effects of DT compared with any control group (positive control or no intervention) on global cognitive function, specific cognitive subdomains, mental health, and quality of life in older adults with MCI. Furthermore, we explored whether different measurement instruments, types of dances, and duration and frequency of interventions affect the effectiveness of DT.

## Methods

### Design

This systematic review and meta-analysis were conducted in strict accordance with the Preferred Reporting Items for Systematic Reviews and Meta-Analyses (PRISMA) guidelines [19] (Additional file 1). The study protocol was registered in PROSPERO (Number: CRD42018096085).

### Data sources and search methods

The following English and Chinese electronic databases were searched: PubMed, Embase, Cochrane Central Register of Controlled Trials, Cumulative Index to Nursing and Allied Health Literature, Web of Science, PsycINFO, ProQuest Dissertations &Theses, Scopus, Taiwanese Airiti Library, Chinese National Knowledge Infrastructure, Chinese Wanfang database, China Science and Technology Journal Database, and Chinese Biomedical Literature Database. Literature published from inception up to September 23, 2023, was checked. We used a combination of subject terms and free words to develop the search strategy. Appropriate search queries were developed based on the characteristics of each database. The full search strategies for all databases are available in Additional file 2. Additional searches were conducted using Google Scholar to identify unpublished or grey literature.

### Eligibility criteria and study selection

All retrieved documents were first de-duplicated using Endnote X9 and manual screening. Two reviewers independently identified studies that potentially met the inclusion criteria by reading the titles and/or abstracts of the articles for inclusion. Disagreements between two reviewers were resolved by a third reviewer. The inclusion and exclusion criteria were as follows:

1. Study design: Both randomized controlled trials (RCTs) and clinical controlled trials (CCTs) written in English and Chinese were included in this review. Non-experimental studies, protocols, and reviews were excluded.
2. Population: Older adults (aged ≥ 55 years) diagnosed with MCI at baseline were eligible for inclusion. The decision to focus on the age range of 55 years and above was influenced by the increasing prevalence of MCI with advancing age. Additionally, considering that MCI represents a transitional stage between normal aging and mild dementia, early intervention becomes notably crucial, and an expanding body of research is specifically targeting the age group of 55 years and above [20, 21]. Therefore, our choice was based on a specific emphasis on the study population and our research objectives. The diagnosis of MCI was established using the Petersen criteria [22], revised Petersen criteria [23, 24], Matthews criteria [25], a Clinical Dementia Rating score of 0.5 [26], the National Institute on Aging-Alzheimer’ Association core clinical criteria [27], or a combination of these. There were no restrictions on the type of residence (e.g., community living, home living, or nursing home), sex, or ethnicity. Samples with varying levels of cognition (e.g., MCI and dementia patients; MCI and healthy older adults), were excluded.
3. Intervention: Participants in the intervention group exclusively received dance interventions, without any restrictions on the frequency, type, duration, or location of these interventions. Frequency was quantified by considering both the length of individual dance sessions and the number of classes conducted per week, while type referred to the specific dance genre taught in each study. Duration encompassed the overall intervention period.
4. Comparator: Any control group (e.g., no treatment control, waitlist control, or active control).
5. Outcomes: Any of the outcomes in global cognitive function, specific cognitive subdomains, quality of life, and mental health.

### Data extraction

Relevant key information was extracted by one researcher according to a pre-designed data extraction form, while a second researcher reviewed the data. Any disagreements were resolved through discussion or with the assistance of a third researcher. Missing data were requested from the authors, as applicable. Extracts included author name, publication year, country, study design, setting, participant demographics (e.g., the total number of participants, number of males and females, and age), intervention details (e.g., frequency, type, duration, and follow-up), indicators of feasibility (e.g., dropout rate, attendance rate, and adverse events) and outcome measures (e.g., measurement instruments and assessment times).

### Quality assessment

Two reviewers independently used the Risk of Bias 2.0 (RoB 2.0) and the risk of bias in non-randomized studies-of interventions to evaluate the potential for bias in RCTs and CCTs from different aspects [28, 29]. To guide the reviewer’s judgement, the assessment tool sets out multiple signal questions for the evaluation of each module and provides details on the parameters that should be considered when answering each signal question. Moreover, two reviewers judged the overall risk of bias for each study based on the combination of the modules. Disagreements were resolved by a third reviewer.

### Data synthesis and analysis

The STATA 16.0 software was used for the meta-analysis. Pooled standardized mean difference (SMD) of the experimental group and the control group was determined using random- and fixed-effects models. The SMD and 95% confidence intervals (CIs) were reported. The heterogeneity between study results was determined using *I*^*2*^ statistics (*I*^*2*^ > 50% indicates moderate-to-substantial heterogeneity). In the absence of significant heterogeneity, a fixed-effects model (*p* > .05 and *I*^*2*^ < 50%) was used; conversely, a random-effects model (*p* < .05, *I*^*2*^ > 50%) was adopted, and sources of heterogeneity were sought through subgroup analysis. Additional subgroup analyses were performed on the effect of dance on results with regard to the measuring instrument, dance type, and duration and frequency of dance interventions (if applicable). In addition, sensitivity analysis was used to judge the stability of outcome indicators, and the differences between pooled effect sizes were compared mainly by eliminating the included studies on an item-by-item basis. Funnel plots were used to examine publication bias (Additional file 3). Furthermore, Egger’s regression test [30] was conducted to visualize and evaluate the funnel plots, and a statistically significant difference (*p* < .05) denoted the presence of publication bias. A narrative synthesis was performed in case of insufficient data for meta-analysis.

## Results

### Search outcome

**Fig. 1:**
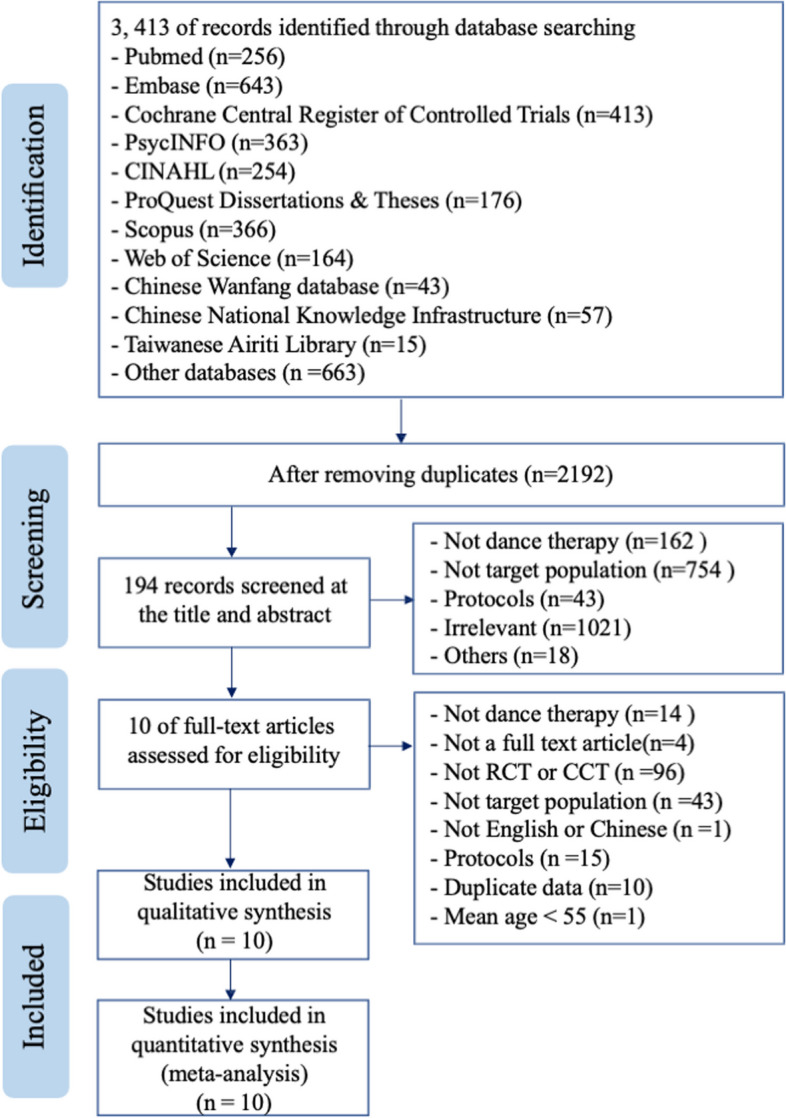
PRISMA flow diagram. CINAHL, Cumulative Index to Nursing and Allied Health Literature; PRISMA, Preferred Reporting Items for Systematic Reviews and Meta-Analyses

A total of 3,413 records were retrieved from the databases and manual searches. Of those, 1,221 duplicate records were removed using Endnote X9, and 1,998 records were excluded after reading the titles and abstracts. Subsequently, 194 articles were selected for full-text screening; of those, 10 studies that met the inclusion criteria were eventually included in the review. The PRISMA flow diagram is illustrated in Fig. 1.

### Included study characteristics

Seven RCTs and three CCTs published between 2016 and 2021 in English and Chinese were included in the systematic review. Studies were conducted in Spain [31, 32], the Philippines [33], Japan [34], Greece [35], and China [36–40]. These studies involved a total of 984 older adults (mean age range: 65–81 years) with MCI (defined based on the Peterson criteria) who were recruited from the community (*n* = 4), nursing homes (*n* = 3), and clinical settings (*n* = 3).

The types of dance used in the intervention group were mostly closely related to the local folklore and socio-cultural context, with five studies involving ballroom dance [31, 33–35, 38], three studies involving square dance [37, 39, 40], and two studies involving aerobic dance [32, 36]. All studies were conducted in groups with a focus on social participation. The duration of the intervention ranged 12–48 weeks and consisted of 1–3 weekly sessions of 30–60 min. Typical sessions followed a ‘low-high-low’ pattern in intensity and were divided into three phases, namely warm-up, dance, and cool-down. The control group consisted of music, physical therapy, health education, and usual care. One study [31] included a control group of participants who were waitlisted. All included studies focused on outcome indicators related to cognitive functioning. Moreover, nine studies addressed indicators of mental health, and five studies addressed indicators of quality of life. Only two studies [31, 32] reported results for 12 and 16 weeks of follow-up, respectively. The summary of study characteristics and outcome measures are presented in Tables 1 and 2.

**Table 1:** Summary of study and dance intervention characteristics for included studies

Author (year) Country	Design	Setting	Participant (Experimental / Control)			Intervention			Control
			Size	Age	Female	Type of dance	Frequency	Duration	
Aguiñaga (2017) [31] Spain	RCT	Adult wellness center	10 11	76.0 (± 6.0) 74.9 (± 6.8)	8 (80.0%) 8 (72.7%)	Latin dance	60 min 2/week	4 months	Waiting list
Doi (2017) [34] Japan	RCT	Community	67 67 67	75.7 (± 4.1) 76.0 (± 4.9) 76.2 (± 4.6)	34(50.7%) 31(46.3%) 39(58.2%)	Ballroom dance	60 min 1/week	10 months	Music program Health education
Lazarou (2017) [35] Greece	RCT	Greek Alzheimer Association and Related disorders	74 65	65.89(± 10.67) 67.92(± 9.47)	53(71.6%) 48(73.8%)	International Ballroom Dancing	60 min 2/week	10 months	Blank control (maintain usual lifestyle)
Dominguez (2018) [33] Philippines	CCT	Community	101 106	68.8 (± 5.6) 69.4(± 6.1)	85 (84.2%) 79 (74.5%)	Ballroom dance	60 min 2/week	12 months	Blank control (maintain usual lifestyle)
Wu (2019) [36] China	RCT	Hospital	29 31	70.3 (± 6.7) 69.0(± 7.3)	15(51.7%) 21(67.7%)	Aerobic dance	35 min 3/week	3 months	Health education
Wang (2020) [40] China	CCT	Nursing home	33 33	81.06 (± 5.17) 81.09(± 7.44)	26(78.8%) 21(63.6%)	Square dance	40 min 3/week	3 months	Blank control (maintain usual lifestyle)
Bisbe (2020) [32] Spain	RCT	Hospital	18 18	72.88 (± 5.60) 77.29(± 5.16)	8(47.1%) 9(50.0%)	Choreography	60 min 2/week	3 months	Physical therapy
Zhao (2020) [37] China	CCT	Community	31 32	73.35(± 5.10) 71.25(± 6.73)	26(83.37%) 26(81.25%)	Square dance	60 min 3/week	3 months	Health education
Fei (2020) [38] China	RCT	School gymnasium	41 41	Not reported	Not reported	International Ballroom Dancing	60 min 3/week	6 months	Health education
Chang (2021) [39] China	RCT	Nursing home	62 47	76.56(± 3.60) 75.94(± 3.61)	Not reported	Square dance	30 min 3/week	4.5 months	Blank control (maintain usual lifestyle)

Age = years and (±standard deviation)

**Table 2:** Summary of feasibility of experimental group and outcome measures for included studies

Author (year) Country	Feasibility of experimental group			Outcome measures									Assessment time
	Adherence	Dropout rate	Adverse events	Global Cognition	Memory	Attention	Executive function	Processing speed	Visuospatial Function	Language	Mental health	Quality of life	
Aguiñaga (2017) [31] Spain	85.00%	None	None	MMSE	Logical Memory I and II	DST SDMT	TMT-B Stroop	TMT-A SDMT		Word fluency	GDS-15	QoL-AD, SF-LLFDI	At baseline, 2 months, 4 months, 6 months, and 8 months
Doi (2017) [34] Japan	86.00%	17.91%	None	MMSE	Story memory, Word list memory		TMT-B	TMT-A					At baseline and post-intervention
Lazarou (2017) [35] Greece	Not reported	10.81%	Not reported	MoCA MMSE	RBMT1, RBMT2, RAVLT	TEA			ROCFT-copy, ROCFT-delay	FAS	GDS-30 NPI		At baseline and post-intervention
Dominguez (2018) [33] Philippines	88.80%	17.40%	None	MoCA						BNT	GDS-30 NPI		At baseline and post-intervention
Wu (2019) [36] China	Not reported	6.90%	None	MoCA MMSE	WMS-RLM	DST SDMT	TMT-B	TMT-A SDMT		Word fluency	GDS-15	SF-36	At baseline, 3 months, and 6 months
Wang (2020) [40] China	80.29%	6.06%	None	MoCA MMSE							GDS-15	SF-12	At baseline, 1.5 months, and 3 months
Bisbe (2020) [32] Spain	95.10%	5.00%	Not reported	MMSE	WMS-III, RBANS		LVF	TMT-A	JLO	CVF	HADS-depression HADS-anxiety	SF-36	At baseline and post-intervention
Zhao (2020) [37] China	Not reported	12.90%	None	MoCA-P							GDS-30		At baseline and post-intervention
Fei (2020) [38] China	Not reported	Not reported	Not reported	MoCA MMSE			TMT-B	TMT-A					At baseline and post-intervention
Chang (2021) [39] China	Not reported	13.89%	Not reported	MoCA							GDS-15	SF-12	At baseline, 9 weeks, and 18 weeks

*Abbreviations: BNT *Boston Naming Test, *CCT *Clinical Controlled Trial, *CVF *Category Verbal Fluency, *DST *Digit Span Test, *FAS *Verbal Fluency F-A-S Test, *GDS-15 *15-item Geriatric Depression Scale, *GDS-30 *30-item Geriatric Depression Scale, *HADS *Hospital Anxiety Depression Scale, *JLO *Judgement of Line Orientation Test, *LVF *Letter Verbal Fluency, *MMSE *Mini-Mental State Examination, *MoCA *Montreal Cognitive Assessment, *NPI *Neuropsychiatric Inventory, *QoL-AD* Quality of Life-Alzheimer’s Disease, *RAVLT *Rey Auditory Verbal Learning Test, *RBANS *Repeatable Battery for the Assessment of Neuropsychological Status, *RBMT *Rivermead Behavioral Memory Test, *RCT *randomized controlled trial, *ROCFT *Rey–Osterrieth Complex Figure Test, *SDMT *Symbol Digit Modalities Test, *SF-12 *12-Item Short Form Health Survey, *SF-36 *36-Item Short Form Health Survey, *TEA *Test of Everyday Attention, *TMT-A *Trail Making Test-A, *TMT-B *Trail Making Test-B, *WMS-III *Wechsler Memory Scale 3rd Edition, *WMS-RLM *Logical Memory subset from Revised Wechsler Memory Scale

Nine studies [31–37, 39, 40] reported the participant dropout rate, which is defined as the percentage of participants who withdrew or discontinued their participation in the intervention before its completion. The dropout rates of these studies were all less than 20%. Reasons for discontinuation of the dance intervention included health-related problems (e.g., back pain, knee pain and other illnesses) [31, 33, 34, 36, 39, 40], family problems [35, 36], leaving the country [31, 33], joining other activity groups [37], and declining to continue for personal reasons [32, 33, 37, 39]. The attendance rate refers to the percentage of participants who attended the scheduled intervention sessions or classes. Five studies [31–34, 40] reported on adherence to the intervention, with rates ranging from 80.29 to 95.10% attendance. Reasons for missed dance classes included illness [31, 33, 34], course difficulty [31], and scheduling conflicts [31]. Six studies [31, 33, 34, 36, 37, 40] also reported on adverse events, defined as any unexpected or harmful events that participants may have experienced during the intervention. However, there were no adverse events reported in any of these studies. The summary of the study feasibility is presented in Table 2.

### Risk of bias and quality assessment

The quality of RCTs was rigorously assessed based on five domains using the RoB 2.0 tool. Two studies [32, 34] were linked to an overall low risk of bias. One study [36] was linked to some concern regarding the risk of bias due to deviations from intended intervention, missing outcome data, and selection of the reported result. Another study [31] was linked to some concern regarding the risk of bias, arising from the selection of the reported result. Furthermore, three studies [35, 38, 39] were linked to an overall high or some concern regarding the risk of bias owing to deviations from the intended intervention. All seven studies were linked to a low risk of bias regarding the randomization process and measuring outcomes. For the CCTs, all studies were linked to a low risk of bias with respect to the selection of participants, classification of interventions, and measuring outcomes. Details on the risk of bias are presented in Additional file 4.

### Study outcomes

#### Meta-analysis of the effect of DT on cognitive function

The meta-analysis indicated that DT had a highly significant effect on global cognitive function (SMD = 0.94; 95% CI: 0.57, 1.30), as well as on memory (SMD = 0.50; 95% CI: 0.33, 0.68), executive function (SMD = − 0.34; 95% CI: −0.56, − 0.12), attention (SMD = 0.33; 95% CI: 0.09, 0.57), and language domain (SMD = 0.42; 95% CI: 0.22, 0.63). However, no beneficial effects were found in processing speed (SMD = − 0.22, 95% CI: −0.59, 0.15) and visuospatial ability (SMD = 0.35; 95% CI: −0.35, 1.06). There was substantial heterogeneity across the studies for global cognitive function (*I*^*2*^ = 88%, *p* < .001), processing speed (*I*^*2*^ = 65%, *p* = .007), and visuospatial ability (*I*^*2*^ = 69.4%; *p* = .071). Low heterogeneity among the studies was observed for memory (*I*^*2*^ = 0%, *p* = .742), executive function (*I*^*2*^ = 0%, *p* = .604), attention (*I*^*2*^ = 11.4%, *p* = .341), and language domain (*I*^*2*^ = 0%, *p* = .759). The effect of DT on specific cognitive subdomains of older adults with MCI is depicted in Additional file 5.

#### Subgroup analyses of the effect of DT on global cognitive function

##### Effect of the measurement instrument on global cognitive function

**Fig. 2:**
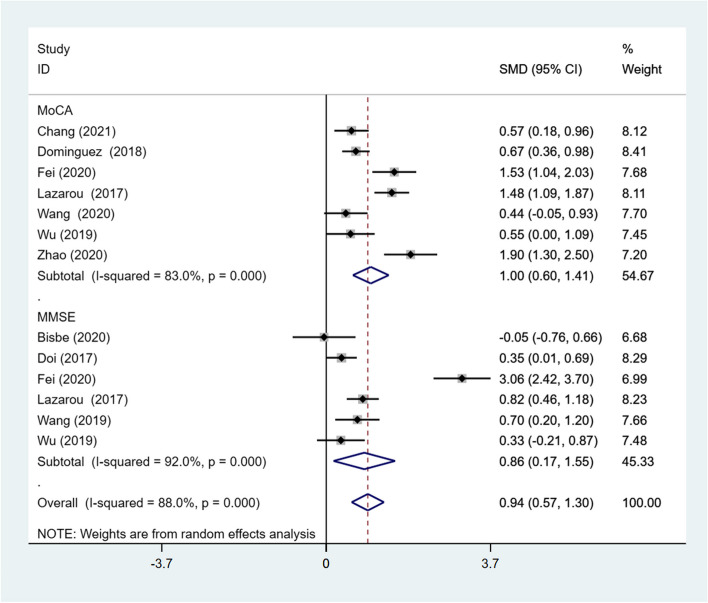
Results of subgroup analysis of global cognitive function according to measurement instrument. CI, Confidence Interval; MMSE, Mini-Mental State Examination; MoCA, Montreal Cognitive Assessment; SMD, Standardized Mean Difference

Subgroup analysis was conducted to compare the interaction between two subgroups using different measurement instruments. The Mini-Mental State Examination (MMSE) and Montreal Cognitive Assessment (MoCA) were adopted to assess global cognitive function among the included studies. Subgroup analysis showed that the effect size of the MoCA subgroup (SMD = 1.00; 95% CI: 0.60, 1.41) was slightly larger than that of the MMSE subgroup (SMD = 0.86; 95% CI: 0.17, 1.55). The effect sizes for both subgroups were statistically significant, and high heterogeneity was observed in both groups (*I*^*2*^ = 83%,* p* < .001; *I*^*2*^ = 92%,* p* < .001) (Fig. 2).

##### Effect of dance type on global cognitive function

**Fig. 3:**
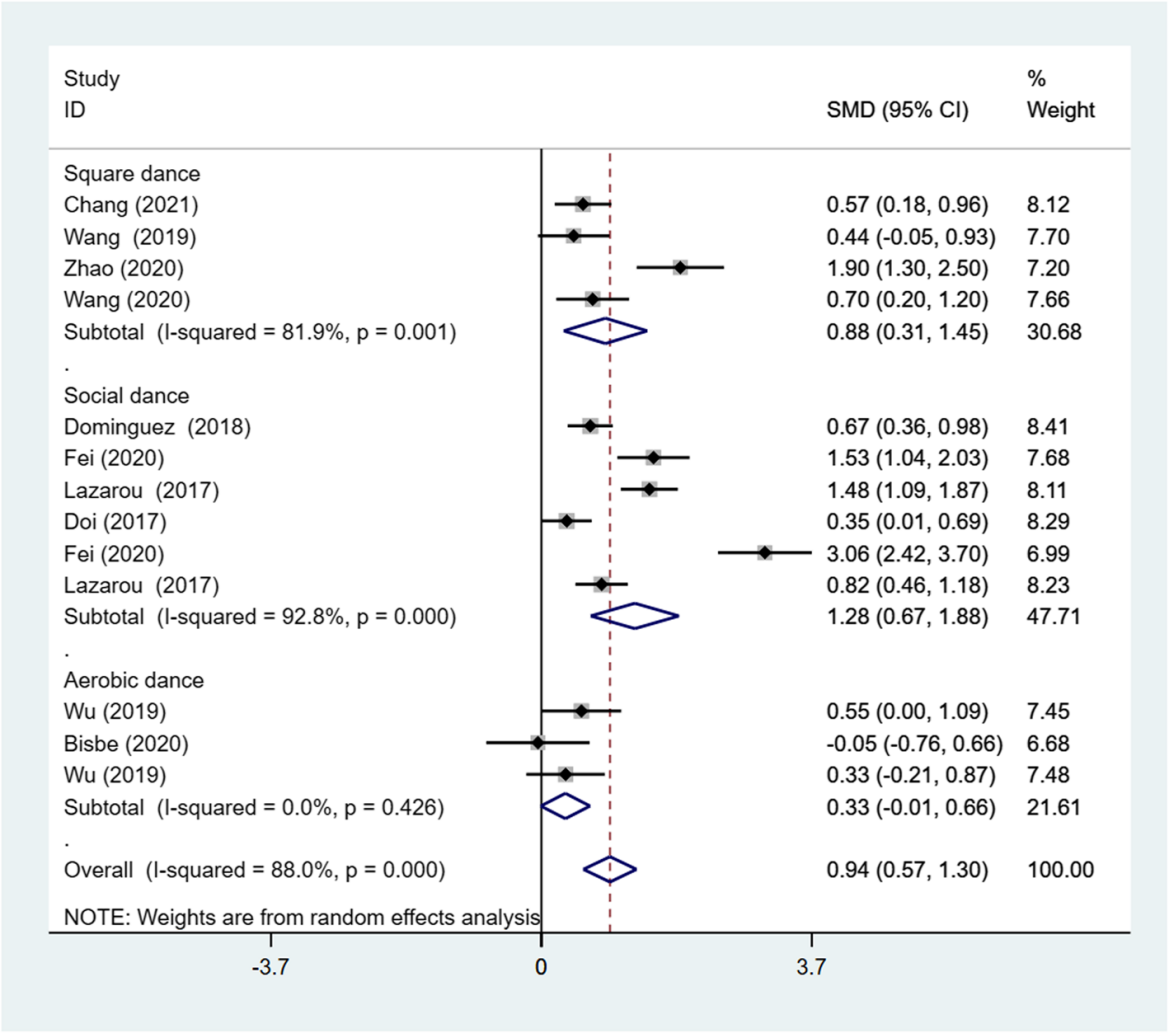
Results of subgroup analysis of global cognitive function according to dance type. CI, Confidence Interval; SMD, Standardized Mean Difference

Subgroup analysis was carried out using dance type (e.g., social dance, aerobic dance, and square dance) as a classification indicator. The meta-analysis employing a random-effects model revealed highly statistically significant differences in global cognitive function compared with the control group for social dance (SMD = 1.28; 95% CI: 0.67, 1.88) and square dance (SMD = 0.88; 95% CI: 0.31, 1.45). Additionally, the effect of aerobic dance was not significant (SMD = 0.33; 95% CI: −0.01, 0.66), and there was no heterogeneity observed among the included studies (*I*^*2*^ = 0%, *p* = .426). Nevertheless, there was considerable heterogeneity of effect sizes for global cognitive function (*I*^*2*^ = 88%, *p* < .001) (Fig. 3).

##### Effect of intervention duration and frequency on global cognitive function

Subgroup analyses were conducted using intervention duration (≤ 12 weeks or > 12 weeks) and intervention frequency (< 3 times/week or ≥ 3 times/week) as subgroup indicators.

**Intervention duration**

**Fig. 4:**
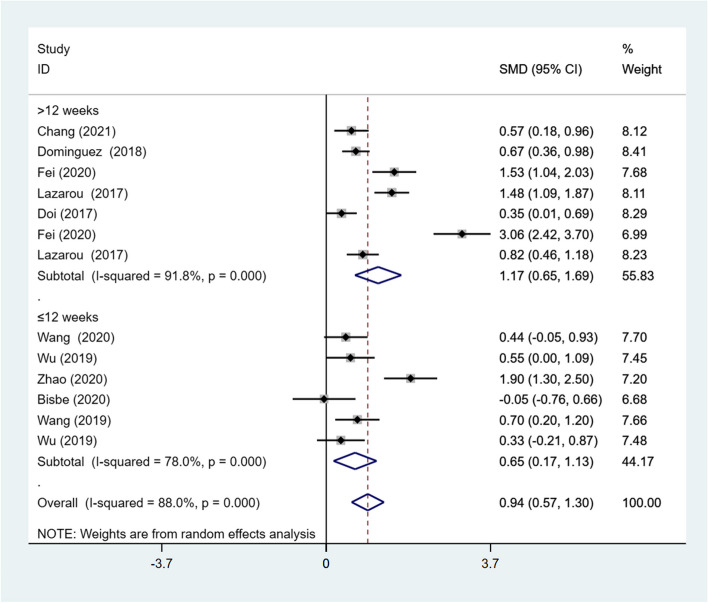
Results of subgroup analysis of global cognitive function according to intervention duration. CI, Confidence Interval; SMD, Standardized Mean Difference

Four studies all conducted dance interventions for 12 weeks, and five studies conducted dance interventions for 18–48 weeks. The subgroup analysis revealed that DT had a significant impact on the global cognitive function of older adults with MCI, regardless of the intervention duration (SMD = 0.94; 95% CI: 0.57, 1.30). There was substantial heterogeneity in both subgroups (*I*^*2*^ = 91.8%, *p* < .001; *I* ^*2*^ = 78.0%, *p* < .001). Nevertheless, the effect size of the intervention duration > 12 weeks (SMD = 1.17; 95% CI: 0.65, 1.69) was larger than that of duration ≤ 12 weeks (SMD = 0.65; 95% CI: 0.17, 1.13) (Fig. 4).

**Intervention frequency**

**Fig. 5:**
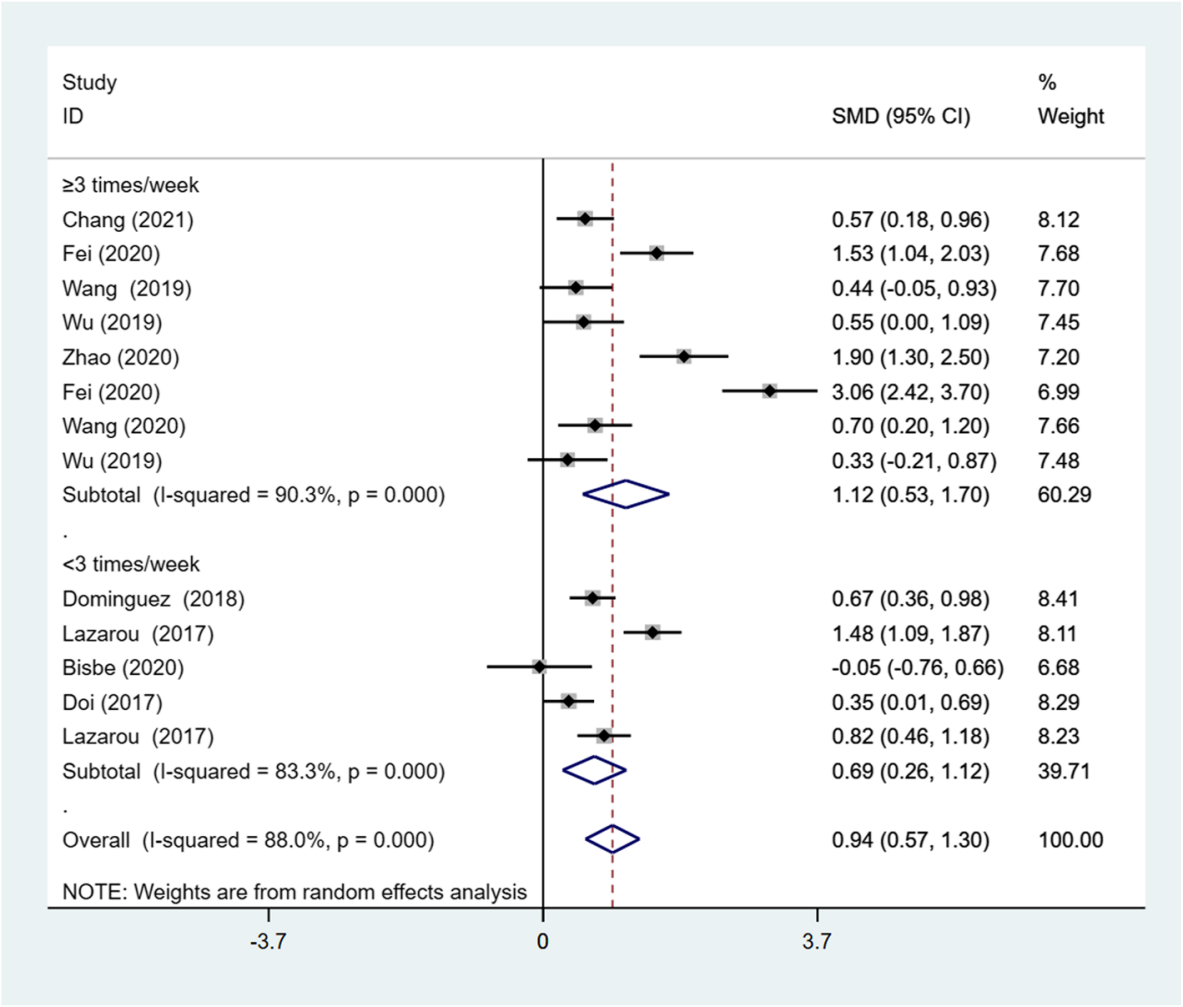
Results of subgroup analysis of global cognitive function according to intervention frequency. CI, Confidence Interval; SMD, Standardized Mean Difference

Five studies had an intervention frequency of ≥ 3 times per week, and four studies had an intervention frequency of < 3 times per week. The meta-analysis using a random-effects model demonstrated that DT had a significant effect on global cognitive function regardless of the intervention frequency (SMD = 0.94; 95% CI: 0.57, 1.30). Moreover, the intervention frequency ≥ 3 times per week (SMD = 1.12; 95% CI: 0.53, 1.70) has a greater effect size than < 3 times per week (SMD = 0.69; 95% CI: 0.26, 1.12). However, the heterogeneity was substantial for these two subgroups (*I*^*2*^ = 90.3%, *p* < .001; *I*^*2*^ = 83.3%, *p* < .001) (Fig. 5).

#### Effect of DT on mental health

**Fig. 6:**
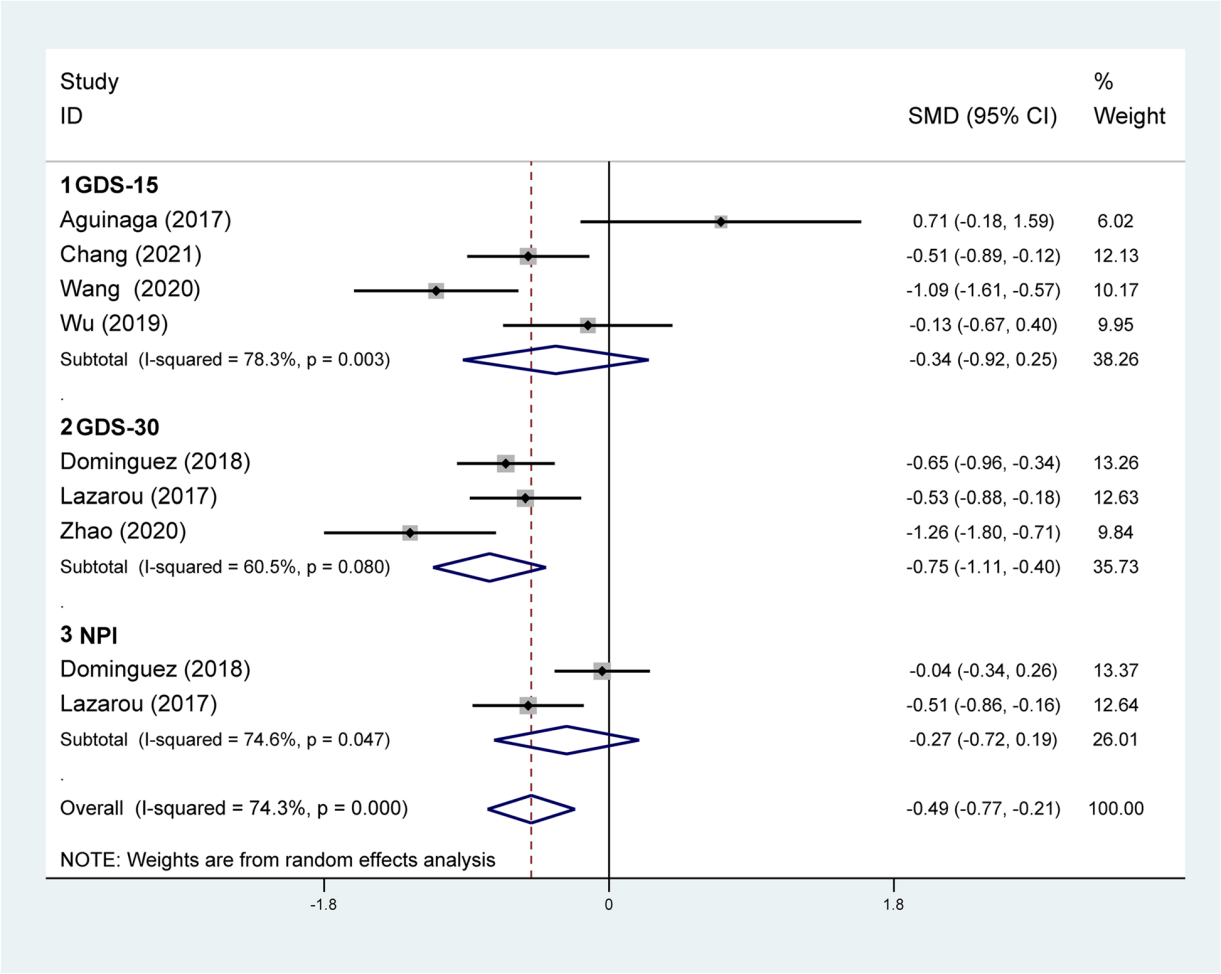
Results of subgroup analysis of mental health according to measurement instrument. CI, Confidence Interval; GDS, Geriatric Depression Scale; NPI, Neuropsychiatric Inventory; SMD, Standardized Mean Difference

Mental health is considered as the well-being of an individual’s spiritual dimension. Related outcomes, such as depression and neuropsychiatric symptoms, were reported in seven studies involving 613 participants. According to the meta-analysis, DT showed significantly greater improvement in mental health compared with the control group (SMD = − 0.49; 95% CI: −0.77, − 0.21). However, there was a high heterogeneity in the effect sizes (*I*^*2*^ = 74.3%, *p* < .001) (Fig. 6).

#### Effect of DT on quality of life

**Fig. 7:**
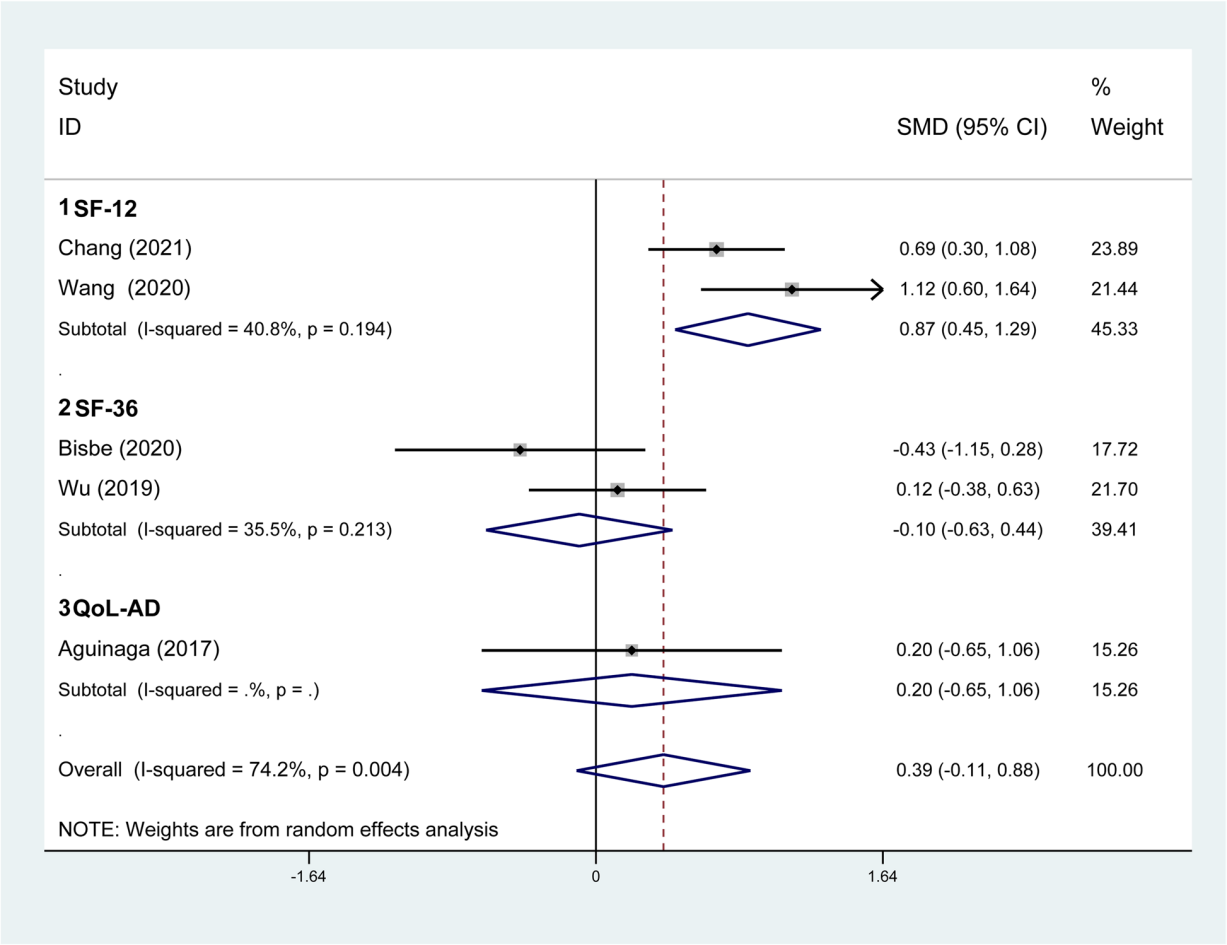
Results of subgroup analysis of quality of life according to measurement instrument. CI, Confidence Interval; QoL-AD, Quality of Life-Alzheimer’s Disease; SF-12, 12-Item Short Form Health Survey; SF-36, 36-Item Short Form Health Survey; SMD, Standardized Mean Difference

Five studies examined the effect of DT on quality of life using the 12-Item Short Form Health Survey (SF-12), 36-Item Short Form Health Survey (SF-36), and Quality of Life-Alzheimer’s Disease (QoL-AD). A total of 287 participants were pooled for meta-analysis using the random-effects model. The results demonstrated that DT did not exhibit a significant improvement in quality of life (SMD = 0.39; 95% CI: −0.11, 0.88). In addition, considerable heterogeneity was observed among the five studies (*I*^*2*^ = 74.2%, *p* = .004). The subgroup meta-analysis using the measurement instrument as a classification indicator showed that the SF-12 subgroup (SMD = 0.87; 95% CI: 0.45, 1.29) was associated with significantly higher efficacy versus the SF-36 (SMD = − 0.10; 95% CI: −0.63, 0.44) and QoL-AD subgroups (SMD = 0.20; 95% CI: −0.65, 1.06). Only the SF-12 subgroup reached statistical significance in terms of effect size. Low heterogeneity was observed for both SF-12 (*I*^*2*^ = 40.8%, *p* = .194) and SF-36 (*I*^*2*^ = 35.5%, *p* = .213) subgroups (Fig. 7).

### Sensitivity analysis and publication bias

A sensitivity analysis was performed on global cognitive function, and mental health. After excluding each study on an item-by-item basis, changes in total effect sizes were evaluated, with effects remaining largely unchanged. Furthermore, a sensitivity analysis was conducted on quality of life. Following the exclusion of one study [32], the results suggested a significant impact of DT on improvements in quality of life; these findings contradicted the previous results. Egger’s regression tests were not significant for any of the outcome indicators, suggesting absence of publication bias (*p* > .05) (Additional file 6).

## Discussion

This review found that DT significantly improved global cognitive function, specific cognitive subdomains (i.e., memory, executive function, attention, language), and mental health (i.e., depression and neuropsychiatric symptoms). Controversy remains on the role of DT in improving processing speed, visuospatial ability, and quality of life in older adults with MCI.

This systematic review suggested that DT significantly improved global cognitive function in older adults with MCI, consistent with a previous meta-analysis [7]. The possible mechanism underlying this effect may be the induction of neuroplasticity by DT across seven neurobehavioral domains, namely sensory, motor, cognitive, social, emotional, rhythmic, and creative [41]. The process of engaging in DT requires a complex integration of multiple sensory channels and fine motor control. Therefore, it may have strong and lasting effects on global cognitive function [41]. Furthermore, Trost et al. [42] demonstrated that different music genres influence brain processes, such as reward, memory, reflexivity, and sensorimotor processing, including activation of the anterior ventricular prefrontal cortex and hippocampus. Therefore, as a music-based physical and mental activity, dance may induce similar structural and neuroplastic changes in the brain [43].

Impaired memory function is the primary observable symptom in older adults with MCI [3]. Encouragingly, our results showed that DT was associated with a significant improvement in memory, which is consistent with the findings of previous systematic reviews [7, 11, 12]. Dance practice involves the complex cognitive process of learning and remembering dance sequences or movements, requiring the involvement of different somatosensory and cognitive brain areas [41]. In recent years, a growing number of studies have evaluated the effects of dance on the structure and function of the brain. A study assessing the effects of a 3-month aerobic dance intervention in older adults with MCI revealed significant improvements in fractional anisotropy value in the white matter fiber tracts of the cingulate fasciculus bilaterally, the left hippocampus of the cingulate fasciculus, and the left superior longitudinal fasciculus in the participants; notably, these areas are closely related to memory and cognitive function [44], consistent with the findings of another study [45]. The hippocampus is the central structure of the limbic system and plays a role in short- and long-term memory and spatial orientation [46]. Hence, hippocampal plasticity may mediate the effects of DT on memory improvement in older adults with MCI.

Our results showed that DT significantly enhanced executive function among older adults with MCI. Executive function refers to the ability of individuals to effectively initiate and complete purposeful activities. This is a complex cognitive process involving planning, initiation, sequencing, cognitive flexibility, feedback, decision-making, and judgement [47]. Previous studies have found that DT was effective in enhancing cognitive flexibility in older adults [36]; our study yielded similar results. The involvement of the prefrontal lobe and the impairment of communication function between various cerebral regions in older adults with MCI may be important for the decline of executive function. Recent work indicated that participants who underwent a 6-month intervention of aerobic dance experienced significant improvements in executive function [48]. This improved performance may be the result of structural and functional changes in the prefrontal cortex associated with the dance intervention, as reflected by the greater grey matter volume and higher activation in prefrontal regions [49, 50].

It had been shown that DT improves attention in older adults [51], and this positive effect was also noted in the present study. The dance process involves complex movement sequences; this means that participants need to focus their attention on observing and remembering the dance sequences to perform the correct movements [16]. The distributed neural network involving the frontal lobe, parietal lobe, and temporal lobe reflects the brain activities related to attention control [52]. A study revealed that dancers showed stronger neural activity in the middle frontal gyrus and inferior frontal gyrus than non-dancers; these areas are closely associated with attentional control [53]. This may provide a preliminary explanation for the improvement in attention performance induced by DT. In addition, several previous systematic reviews did not yield conclusive results with regard to the beneficial effect of DT on attention, contradicting the findings of this study [7, 11]. Therefore, more RCTs with large sample sizes are warranted to further validate the effects of dance on attention.

Our study indicated that DT had benefits on language, but no significant effect on visuospatial ability and processing speed. The improvement observed in language in the DT group could be due to verbal interaction which occurs during group dance sessions. DT is essentially a social activity which provides a supportive environment that maximizes the retention of participants’ communication skills [16, 54]. In contrast with the insignificant effect detected in our study, previous reviews had identified potential benefits of DT on visuospatial ability [12, 51, 55]. A recent RCT suggested that participation in a 10-month systematic dance session may improve the visuospatial skills of older adults with MCI to a greater extent versus control [35]. One possible explanation is that dancers are trained to understand where the body is in space and, consequently, navigate and coordinate movements in space, as well as manage the spatial distance between themselves and others [41]. However, only two of the studies included in our meta-analysis reported findings on visuospatial ability, assessed using different measurement instruments. Additionally, substantial heterogeneity in effect size was observed across studies. These could be among the reasons responsible for the insignificant findings in this study. With regard to processing speed, we reached similar conclusions to the previous meta-analysis [7], which did not find a significant effect of dance on processing speed in older adults with MCI. This may be due to the small number of included studies (only five studies), small sample size, and the use of different measurement instruments. Therefore, more high-quality studies are required to draw credible conclusions on visuospatial ability and processing speed.

Furthermore, this review included subgroup analyses of different intervention characteristics (i.e., measurement instrument, dance type, and duration and frequency of intervention) to identify more effective dance interventions. Consistent with the findings of a previous study [7], the results of the subgroup analysis suggested that differences in measurement tools did not influence the effect of the intervention on overall cognitive function. The included studies were classified into subgroups according to the dance type (i.e., social, aerobic, and square). The type of dance was determined based on the specific content of the study. The results demonstrated that social and square dancing improved global cognitive function; however, a similar positive performance was not observed with aerobic dance. This may be due to the small number of studies (*n* = 3) and the small sample size (*n* = 139) of the aerobic dance subgroup. In addition, there was high heterogeneity in the design of interventions across dance types, including intervention duration and frequency, which may lead to different estimates of effect sizes. Subgroup analysis results identified a significant effect of DT on global cognitive function regardless of the duration and frequency of the intervention. Nonetheless, interventions of longer duration (> 12 weeks) and higher frequency (≥ 3 times per week) showed a larger pooled effect size, in line with previous findings [7]. One possible explanation is that a longer duration of intervention and more frequent interventions can provide more stimulation for participants to maintain the effectiveness of the intervention. However, due to significant differences in the duration and frequency of interventions in the included studies, it was not possible to determine the optimal intervention regimen. Future studies should focus on establishing the optimal intervention regimen in this setting.

In terms of mental health, all seven studies included in this analysis reported depression-related outcomes, while two of them assessed neuropsychiatric symptoms. Depression is a risk factor for cognitive decline and a common manifestation of neuropsychiatric syndrome in older adults with MCI [56, 57]. In contrast with the significant effect recorded in our study, a recent review showed that dance interventions do not produce statistically significant improvements in depression among older adults with MCI [7]. Nevertheless, a series of studies have confirmed the positive effects of dance on depressive states. This may be because dance intervention is a fun music-based physical and mental activity that offers participants a sense of pleasure and relieves depression [37]. Neurobiological studies have demonstrated that an increase in parasympathetic activity, reduction in serum cortisol levels, and suppression of cardiovascular stress response caused by musical stimulation are potential mechanisms contributing to the alleviation of depression in older adults with MCI [13, 58]. Additionally, group settings stimulate social interaction among older individuals and contribute to a reduction in social isolation, loneliness, and depressed mood [41, 54].

Consistent with the results reported in a previous meta-analysis [7], older adults with MCI did not experience an improvement in quality of life after receiving the dance intervention. Surprisingly, through subgroup analysis, we found different effects of measurement tools on the quality of life. This review showed a significant effect on quality of life when measured with SF-12, but not with SF-36. The different results may be attributed to a large difference between the sample size measured by SF-12 (*n* = 175) and that measured by SF-36 (*n* = 91). In addition, these discrepancies may be caused by differences in the number of entries for the measurement instruments. Compared with the SF-36, the SF-12 is a shorter questionnaire, thereby imposing less burden on the study participants; hence, it is likely that the results are more reliable [59]. Sensitivity analysis revealed that the effect of DT on quality of life was statistically significant following the exclusion of one study [32]. A possible explanation is that the positive control group in the excluded study received physiotherapy, and the beneficial effect of physiotherapy on quality of life has been confirmed in previous studies [60]. Thus, the dance intervention group did not demonstrate better quality of life than the physiotherapy group. Moreover, the benefits of dance interventions in reducing social isolation have been documented [54]. It is established that reduction of social isolation and strengthening of social connections are essential for improving the well-being and quality of life of older adults. This may explain the improvement in quality of life induced by dance interventions in older adults with MCI.

In addition, this systematic review yielded encouraging results regarding the feasibility indicators of dance interventions. The study outcomes revealed a high level of participant compliance, with dropout rates consistently below 20%. Among the six included studies, no adverse events were reported, indicating the safety of dance interventions. It is noteworthy that four additional studies did not provide descriptions of adverse event reporting, and the absence of reporting in these studies does not necessarily imply the absence of adverse events. These findings highlight the attractiveness of dance interventions as a potential cognitive intervention approach and offer insights for improving intervention feasibility in future research.

## Limitations

This review has some limitations. Firstly, due to the small sample size of some of the included studies, the effectiveness of dance interventions needs to be confirmed and refined in future investigations with larger sample sizes. Secondly, there was substantial heterogeneity in the included studies (e.g., type of dance, measurement tools, and duration and frequency of interventions). Moreover, most subgroup analyses failed to effectively reduce heterogeneity, which may have influenced the effect size of the pooled results. Thirdly, sufficient blinding and allocation concealment methods were not performed in some of the included studies; this may have impacted the validity of the results. Lastly, as the languages of the included studies were limited to English and Chinese, there may be publication bias due to incomplete inclusion.

## Conclusion

This review provided evidence for the positive effects of DT on global cognitive function, specific cognitive subdomains (i.e., memory, executive function, attention, language), and mental health (i.e., depression and neuropsychiatric symptoms). DT is a mind-body activity with the potential to stimulate neuroplasticity, making it a promising complementary treatment for older adults with MCI. However, it’s essential to exercise caution in interpreting these results due to the limited number of included studies and the varying quality of the evidence. Future research should aim to determine the optimal intervention regimen, assess the sustainability of its effects over time, and investigate whether DT yields superior cognitive and mental health-related benefits compared to other non-pharmacological therapies. Additionally, to enhance the robustness of future investigations, more comprehensive tracking of adverse events, detailed reporting of participant attendance and dropout rates, and a thorough examination of the factors facilitating or hindering the engagement and adherence of older adults with mild cognitive impairment in dance interventions should be incorporated into study protocols.

### Supplementary Information

**Additional file 1:** **Supplementary Material 1.** PRISMA 2020 Checklist. **Supplementary Material 2**. Search strategy. **Supplementary Material 3**. Funnel plot. **Supplementary Material 4**. Risk of bias summary (randomized controlled trials). **Supplementary Material 5**. Forest plot of the effect of dance therapy on specific cognitive subdomains. **Supplementary Material 6**. Tests for Publication Bias (Egger's test).

## Data Availability

All extracted data used in this review has been reported in the text, figures, tables, and Additional file.

## Abbreviations

CCTs: Clinical Controlled Trials
CIs: Confidence Intervals
DT: Dance Therapy
MCI: Mild Cognitive Impairment
MMSE: Mini-Mental State Examination
MoCA: Montreal Cognitive Assessment
PRISMA: Preferred Reporting Items for Systematic Reviews and Meta-Analyses
QoL-AD: Quality of Life-Alzheimer's Disease Scale
RCTs: Randomized Controlled Trials
RoB 2.0: Risk of Bias 2.0
SF-12: Health survey questionnaire Short From-12
SF-36: Health survey questionnaire Short From-36
SMD: Standardized Mean Difference
